# Lymphangioleiomyomatosis, multifocal micronodular pneumocyte hyperplasia, and sarcoidosis: more pathological findings in the same chest CT, or a single pathological pathway?

**DOI:** 10.1186/s12890-017-0447-x

**Published:** 2017-07-28

**Authors:** Fabiano Di Marco, Giuseppina Palumbo, Silvia Terraneo, Gianluca Imeri, Elena Lesma, Nicola Sverzellati, Angela Peron, Lorenzo Gualandri, Maria Paola Canevini, Stefano Centanni

**Affiliations:** 1 Respiratory Unit, Ospedale San Paolo, Department of Health Sciences, Università degli Studi di Milano, Via A. di Rudinì, 8, 20142 Milan, Italy; 2 Laboratories of Pharmacology, Università degli Studi di Milano, Milan, Italy; 3 Radiology, Department of Medicine and Surgery, University of Parma, Parma, Italy; 4 Epilepsy Center, Ospedale San Paolo, Department of Health Sciences, Università degli Studi di Milano ASST Santi Paolo e Carlo, Milan, Italy; 5 Dermatologic Clinic, Ospedale San Paolo, ASST Santi Paolo e Carlo, Milan, Italy; 6 Respiratory Unit, Ospedale San Paolo, Department of Health Sciences, Università degli Studi di Milano, Milan, Italy

**Keywords:** LAM, TSC, Sarcoidosis, Thoracic images

## Abstract

**Background:**

Autoimmune hepatitis/primary biliary cirrhosis overlap syndrome, lymphangioleiomyomatosis/tuberous sclerosis complex (LAM-TSC), and sarcoidosis are three rare diseases. Here we present, to the best of our knowledge, the first description of a patient with the coexistence of these three diseases.

**Case presentation:**

A 47-year-old woman affected by LAM-TSC and primary biliary cirrosis/autoimmune hepatitis overlap syndrome. During her follow up a high resolution chest CT scan (HRTC) confirmed the presence of both multiple cysts and micronodular opacities consistent with multifocal micronodular pneumocytes hyperlasia (MMPH), and revealed multiple hilar-mediastinal symmetrical lymphadenopathies suggestive of sarcoidosis. Simultaneously, subcutaneous nodules appeared on her forearm bilaterally. Cutaneous biopsy showed granulomatous dermatitis with sarcoid-like granulomas. A diagnosis of stage I pulmonary sarcoidosis was made. No treatment for sarcoidosis was initiated since the patient had neither systemic involvement, nor respiratory impairment.

**Conclusions:**

The presence of more than one rare disease should challenge the concept of a potential common underlying mechanism, since the a priori probability of the concomitant presence of different conditions with different pathogenic mechanisms - especially if rare diseases - is low.

We speculate that the dysregulation of the pathway involving mTOR and MAPK and their interaction might play a role in the pathogenesis of other diseases, including sarcoidosis.

## Background

Tuberous sclerosis complex (TSC) is a rare genetic disorder, characterized by predominantly benign tumours developing potentially in all organ systems. Pulmonary involvement consists of Lymphangioleiomyomatosis (LAM) and Multifocal Micronodular Pneumocyte Hyperplasia (MMPH), which cause cystic and nodular diseases, respectively. Pneumothorax and chylothorax are common clinical presentations of LAM, whereas MMPH is usually asymptomatic. Here we describe a female with TSC and LAM with new pulmonary findings.

## Case presentation

A 47-year-old woman, affected by TSC with a mutation identified in the *TSC1* gene [c.682C > T (p.Arg228*)], was referred to the TSC Clinic of San Paolo Hospital (Milan, Italy). Family history was noticeable for Addison’s disease and brain glioblastoma (in her mother) and idiopatic pulmonary fibrosis (in her father). Her daughter was affected by TSC. The patient suffered from primary biliary cirrosis/autoimmune hepatitis overlap syndrome. She was treated with ursodeoxycholic acid (15 mg/kg/day).

She received a diagnosis of LAM by chest CT scan, which showed bilateral lung cysts randomly distributed thoughout the lungs. Chest CT scan revealed also the presence of sclerotic bone lesions. Pulmonary function tests were normal, such as the 6-min walking test. Vascular endothelial growth factor (VEGF)-D, a lymphangiogenic growth factor proposed as a biomarker for LAM diagnosis and severity, was 582 pg/mL (normal limits 153–642 pg/mL). Dermatological examination showed hypomelanotic macules, facial angiofibromas, xantelasma palpebrarum, periungual fibromas and erythematous plaque on the left knee. She had a renal angiomyolipoma of 4.2 cm in the left kidney. Brain magnetic resonance (MR) showed the presence of cortical tubers. There was no ocular or heart involvement.

**Fig. 1:**
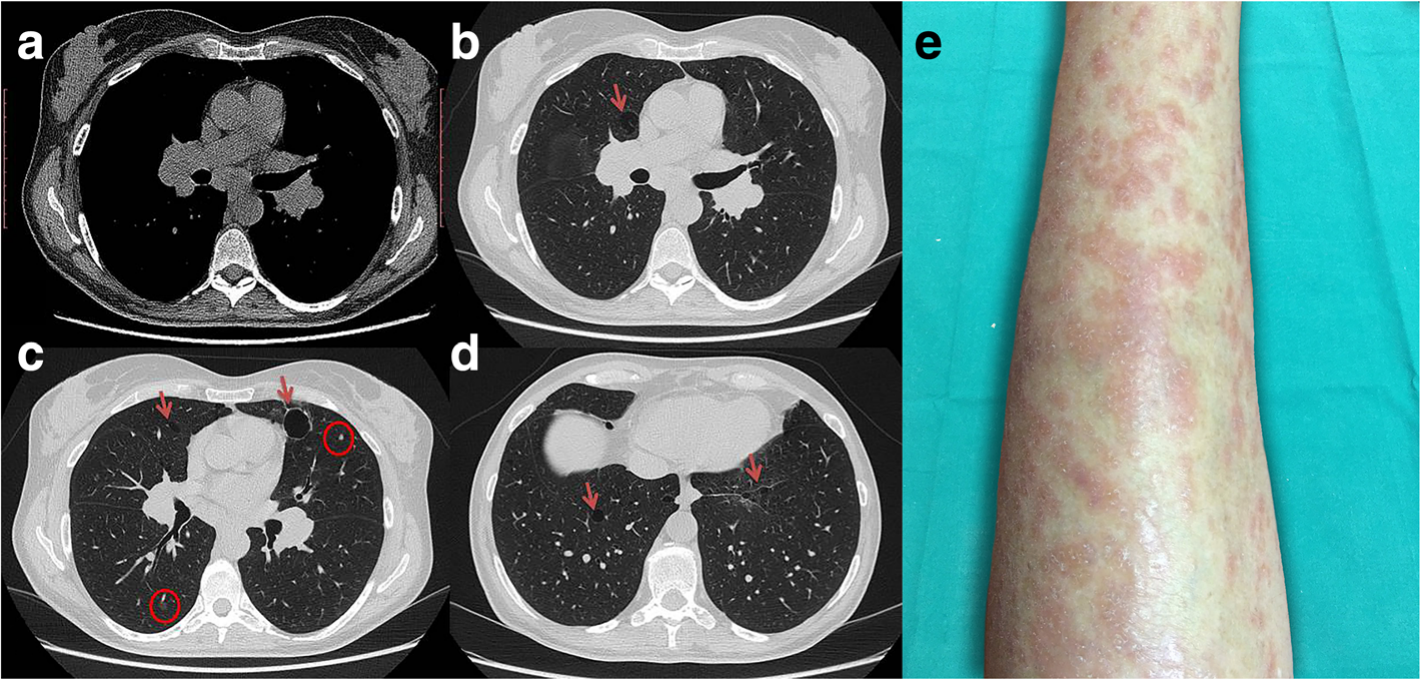
**a** The mediastinal window HRCT image shows symmetrical hilar and subcarinal enlarged lymphnodes. **b**-**d** HRCT images show scattered lung cysts (arrows) consistent with lymphangioleiomyomatosis and either solid or subsolid micronodules (circles). **e** Multiple subcutaneous painless, hard papules and nodules covered by eritematous skin on the left forearm

One year later, the patient was adressed to our clinic. A new high resolution chest CT scan (HRTC) was performed, and confirmed the presence of both multiple cysts and micronodular opacities, consistent with MMPH. Multiple hilar-mediastinal lymphadenopathies were also identified. Of note, hilar lymphadenopathies were symmetrically enlarged (Fig. 1). Simultaneously, subcutaneous nodules appeared on the patient’s forearm bilaterally (Fig. 1e), prompting a biopsy that resulted in the hystopathologic diagnosis of non-necrotizing granulomas, with mono- and multinucleate epithelioid cells, some of them with asteroid bodies surrounded by a sparse lymphocytic infiltrate, suggestive of granulomatous dermatitis with sarcoid-like granulomas. Diseases other than sarcoidosis were ruled out by second line evaluations on histology samples, such as Ziehl-Neelsen, periodic acid-Schiff (PAS), and Giemsa staining techniques. Immunologic studies showed positive antimitochondrial antibody and antinuclear antibody (titre 1:1280, speckled pattern). IgG, IgM and IgA levels resulted within normal range. HIV test was negative. Other causes of granulomatous disease such as drug-induced hypersensitivity, pneumoconiosis, pulmonary histiocytic disorders, diseases associated with vascular inflammation were ruled out through clinical history, examination and the results of instrumental tests.

Thus, a diagnosis of sarcoidosis was considered. In order to evaluate the extent of the disease, laboratory tests were performed and showed normal serum angiotensin conversion enzyme (ACE) and serum and urinary calcium levels. Abdominal ultrasonography showed an enlarged liver with irregular edges and nodular heterogeneous echotexture, with mild steatosis and multiple renal cysts without evidence of nephrolithiasis. Bilateral x-ray of the hands did not show alterations in bone structure. The ophthalmologic examination, including funduscopic evaluation showed no signs of uveitis and confirmed the absence of retinal hamartomas and achromic patches related to TSC. EKG and echocardiography showed no abnormalities. Pulmonary function tests, including spirometry and lung volumes were normal; a mild reduction in diffusing capacity for carbon monoxide was detected. The six-minute walk test was normal. A diagnosis of stage I pulmonary sarcoidosis was made. No treatment for sarcoidosis was initiated since the patient had neither systemic involvement, nor respiratory impairment. A treatment with Sirolimus was not performed due to the limited pulmonary cystic involvement and the lack of respiratory symptoms, and a clinical follow-up was carried on.

## Discussion and conclusions

This case report represents the first description of the coexistence of three rare disorders: autoimmune hepatitis/primary biliary cirrhosis overlap syndrome, TSC/LAM, and sarcoidosis. The presence of more than one rare disease should challenge the concept of a potential common underlying mechanism.

Lymphadenopathy, both thoracic and abdominal, has been described as another possible feature of LAM. For instance, a recent study on 138 patients with LAM, both sporadic and associated with TSC, found a prevalence of 9.4% for mediastinum and pulmonary hilum lymphatic lesions [1]. However, the presence of granulomatous skin lesions has not yet been described in LAM associated with TSC. Thus, the results of the skin biopsy together with the presence of bilateral hilar lymphadenopathy lead to the diagnosis of sarcoidosis.

With respect to sarcoidosis, the importance of host susceptibility and gene-environment interaction is widely accepted [2]. Although sarcoidosis does not meet the criteria for autoimmune disease, it can coexist with a wide range of autoimmune disorders, including primary biliary cirrhosis, which is characterized by hepatic granulomas formation, connective tissue diseases (e.g. systemic sclerosis and Sjogren’s syndrome), Addison’s disease, and thyroiditis [3]. It is noteworthy that the diagnosis of sarcoidosis in case of granulomatous skin lesions is made by exclusion criteria. Granulomatous lesions have been described in case of granulomatous-lymphocytic interstitial lung disease (GLILD) associated with common variable immunodeficiency (CVID), drug toxicity, or infections such as tuberculosis or fungal infections. All the aforementioned diagnoses were ruled out in our patient. As previously stated, the association between granulomatous lesions and autoimmune hepatitis/PBC, such as other immune-mediated and chronic inflammatory disease, has been previously described [4].

TSC is an autosomal-dominant disease caused by heterozygous loss-of-function mutations in the *TSC1* (chromosome 9q34) or *TSC2* (chromosome 16p13) tumour suppressor genes coding for hamartin and tuberin, respectively. Tuberin and hamartin, together with TBC1D7, form a complex that functions as a negative regulator of mammalian target of rapamycin (mTOR) through the inhibition of Rheb. Inactivation of TSC1 or TSC2 results in overactivation of mTOR leading to abnormal cell growth, proliferation, metabolism, and angiogenesis. A common molecular mechanism for LAM/TSC and sarcoidosis is not known. However, a strong immunoreactivity for cathepsin-k was demonstrated in spindle and epithelioid-shaped cells of lung LAM and in granulomas of sarcoidosis cases [5]. Since it is known that modulation of cathepsin-k may occur through the mTOR pathway, it is possible to speculate that the mTOR pathway might play a role in sarcoidosis [6]. Several evidences demonstrated an integration of signaling of the mTOR pathway and mitogen-activated protein kinases (MAPKs)-Erk activation. For instance, vascular endothelial growth factor (VEGF), a lymphangiogenic growth factor present at high levels in serum and urine of patients with LAM, induces phosphorylation of Akt, mTOR, S6 K, S6 and MAPK-Erk. Uncontrolled inflammation and chronic inflammatory diseases may be caused by the persistent activation of MAPKs, which can occur in sarcoidosis [7]. This finding suggests an explanation for the persistent production of several inflammatory cytokines, such as TNF-α and IL-12, in sarcoidosis. Interestingly, Linke et al. found that activation of mTORC1 due to TSC2 deficiency causes granulomatous disease, including sarcoidosis, in both mice and humans. mTORC1 inhibition resolves granulomas in TSC2-deficient mice, suggesting that treatment with mTOR inhibitors, used to stop the progression of benign tumors and LAM in TSC, might improve the signs of sarcoidosis as well [8]. However, the reason why not all patients with TSC develop sarcoidosis is still unknown.

Although at present it is not possible to demonstrate a common mechanism underlying LAM/TSC, sarcoidosis, primary biliary cirrhosis and autoimmune hepatitis, and their coexistence could well occur by chance, we might speculate that the dysregulation of the pathway involving mTOR and MAPK and their interaction may play a role in the alteration of the diseases. Further reports are needed to demonstrate our hypothesis.

## Abbreviations

ACE: Serum angiotensin conversion enzyme
AMA: Antimitochondrial antibody
HRTC: High resolution chest CT scan
LAM: Lymphangioleiomyomatosis
MAPK: Mitogen-activated protein kinases
MMPH: Multifocal micronodular pneumocyte hyperplasia
MR: Magnetic resonance
MTOR: Mammalian target of rapamycin
TSC: Tuberous sclerosis complex
VEGF: Vascular endothelial growth factor
